# Effects of Respiratory Muscle Training on Functional Ability, Pain-Related Outcomes, and Respiratory Function in Individuals with Low Back Pain: Systematic Review and Meta-Analysis

**DOI:** 10.3390/jcm13113053

**Published:** 2024-05-23

**Authors:** Raúl Fabero-Garrido, Iván Rodríguez-Marcos, Tamara del Corral, Gustavo Plaza-Manzano, Ibai López-de-Uralde-Villanueva

**Affiliations:** 1 Department of Radiology, Rehabilitation and Physiotherapy, Faculty of Nursing, Physiotherapy and Podiatry, Complutense University of Madrid, Plaza Ramón y Cajal n° 3, Ciudad Universitaria, 28040 Madrid, Spain; rfabero@ucm.es (R.F.-G.); ivrodr02@ucm.es (I.R.-M.); gusplaza@ucm.es (G.P.-M.); ibailope@ucm.es (I.L.-d.-U.-V.); 2 Instituto de Investigación Sanitaria del Hospital Clínico San Carlos (IdISSC), 28040 Madrid, Spain

**Keywords:** low back pain, respiratory muscle training, respiratory function tests, postural balance, pain threshold, disability

## Abstract

**Objectives**: The aim of this meta-analysis was to determine the effects of respiratory muscle training (RMT) on functional ability, pain-related outcomes, and respiratory function in individuals with sub-acute and chronic low back pain (LBP). **Methods**: The study selection was as follows: (participants) adult individuals with >4 weeks of LBP; (intervention) RMT; (comparison) any comparison RMT (inspiratory or expiratory or mixed) versus control; (outcomes) postural control, lumbar disability, pain-related outcomes, pain-related fear-avoidance beliefs, respiratory muscle function, and pulmonary function; and (study design) randomized controlled trials. **Results**: 11 studies were included in the meta-analysis showing that RMT produces a statistically significant increase in postural control (mean difference (MD) = 21.71 [12.22; 31.21]; decrease in lumbar disability (standardized mean difference (SMD) = 0.55 [0.001; 1.09]); decrease in lumbar pain intensity (SMD = 0.77 [0.15; 1.38]; increase in expiratory muscle strength (MD = 8.05 [5.34; 10.76]); and increase in forced vital capacity (FVC) (MD = 0.30 [0.03; 0.58]) compared with a control group. However, RMT does not produce an increase in inspiratory muscle strength (MD = 18.36 [−1.61; 38.34]) and in forced expiratory volume at the first second (FEV1) (MD = 0.36 [−0.02; 0.75]; and in the FEV1/FVC ratio (MD = 1.55 [−5.87; 8.96]) compared with the control group. **Conclusions**: RMT could improve expiratory muscle strength and FVC, with a moderate quality of evidence, whereas a low quality of evidence suggests that RMT could improve postural control, lumbar disability, and pain intensity in individuals with sub-acute and chronic LBP. However, more studies of high methodological quality are needed to strengthen the results of this meta-analysis.

## 1. Introduction

Currently, cognitive, emotional, and behavioral factors, such as pain catastrophizing and pain-related fear, contribute to the pathophysiology and chronicity of low back pain (LBP) [1,2]. This leads individuals with LBP to adopt safe-seeking practices and movement avoidance [3,4], causing the development of the “disuse syndrome”, which encompasses musculoskeletal decline, impaired coordination, motor control [1], and lumbar segmental instability [5]. Recent meta-analysis findings suggest that LBP reduces the ability to dissociate movement between the trunk and pelvis, increases lumbar paraspinal activation and stiffness, and impairs abdominal postural function [6]. Apart from its respiratory role, the diaphragm and abdominal muscles play a crucial part in spine stabilization [7]. Individuals with chronic LBP often experience diaphragm fatigue [8,9] and struggle to compensate for respiration-related postural sway [10]. Furthermore, the abdominal muscles and lumbar multifidus display a compromised core-stabilizing function, resulting in impaired postural control in individuals with LBP [11]. Therefore, there is a need to explore interventions that target the physical and psychological consequences of disuse syndrome in individuals with LBP.

Clinical practice guidelines highly recommend therapeutic exercise for the treatment of sub-acute and chronic LBP, given that exercise programs are effective in reducing pain and disability and in improving overall recovery, function, and health-related quality of life [12]. Respiratory muscle training (RMT) is a therapeutic exercise modality that improves inspiratory and expiratory muscle strength and respiratory muscle endurance [13]. Recent studies suggest RMT could improve the activation pattern [14] and thickness [15,16] of the diaphragm, transversus abdominis, and lumbar multifidus in individuals with sub-acute and chronic LBP. RMT has been proven to reduce pain intensity [17] and improve respiratory muscle strength [17,18], physical function [17,18], balance [19], and consequently, the overall quality of life [17,18] in several population groups. Finally, recent research highlighted RMT could decrease sympathetic modulation [20], lowering pain intensity levels [21] and psychological distress [22]. Therefore, it seems that the benefits found by RMT in different populations would be of particular interest in individuals with subacute and chronic LBP.

Although several studies have investigated the effects of RMT in individuals with sub-acute and chronic LBP, no systematic review has pooled this evidence. This research is required to reveal whether RMT might have a positive effect on spinal control, respiratory function, and consequently, on LBP symptoms. Given that LBP is a high-burden disease, it is important for physicians and therapists to acquire a complete understanding of the effects of RMT on this population. The objective of this systematic review and meta-analysis was to determine the effects of RMT on functional ability, pain-related outcomes, and respiratory function in individuals with sub-acute and chronic LBP.

## 2. Materials and Methods

This systematic review and meta-analysis followed the criteria of the Preferred Reporting Items for Systematic Reviews and Meta-Analyses (PRISMA) guidelines [23].

### 2.1. Study Selection Criteria

The inclusion and exclusion of the reviewed studies relied on clinical and methodological aspects based on the population–interventions–comparison–outcomes of interest strategy [24] and was registered in PROSPERO (CRD42023387813).

Population: Individuals with >4 weeks of LBP (>3 pain on the visual analogue scale [VAS]) were selected to fit the established standards of sub-acute and chronic LBP [12]. No age or sex limitations were imposed. Data from participants with additional comorbidities were excluded.

Intervention and comparison: The experimental intervention required training the respiratory musculature in terms of strength and/or endurance. Studies employing threshold, resistive loading, or voluntary isocapnic hyperpnea devices and other methods to provide resistance to the respiratory musculature were included. Inspiratory muscle training (IMT), expiratory muscle training (EMT), or combined muscle training (IMT+EMT) modalities were included. Given that the comparisons should permit the extraction of the total effect attributable to RMT, the included studies had to compare RMT versus (1) passive control or (2) sham RMT (without sufficient intensity to generate a training effect). An additional standard intervention could be included if conducted under the same protocol in all study arms. A minimum training period of 4 weeks was required, given that this is the minimum period for physiological adaptations.

Outcomes: The outcomes of interest were as follows: functional ability as evaluated by postural control (center-of-pressure [CoP] path length) and lumbar disability (Athletes Disability Index, Oswestry Disability Index, and Roland–Morris Low Back Pain and Disability Questionnaire); pain-related outcomes measured in terms of pain intensity (VAS, numeric rating scale) and pain-related fear-avoidance beliefs (Fear-Avoidance Beliefs Questionnaire or Tampa Scale of Kinesiophobia [TSK]); and respiratory function as evaluated by measuring inspiratory and expiratory muscle strength (maximal inspiratory and expiratory pressure [MIP and MEP], forced vital capacity (FVC), forced expiratory volume at the first second (FEV1), and the FEV1/FVC ratio.

Study design: Only randomized controlled trials (RCTs) were included. Articles were included if they were published in English, Spanish, or Portuguese.

### 2.2. Search Strategy

The search strategy was performed following the guidelines of Russell-Rose et al. [25]. Searches were conducted in the MEDLINE, Web of Science, Scopus, PEDro, CINHAL, Science Direct, and CENTRAL electronic databases, with no date restrictions, up to 16 December 2022. The search string was adapted to each database, according to the data in Appendix S1. If clinical or methodological doubts arose from potential eligible studies, the authors were contacted by e-mail. Two independent reviewers conducted the search using the same methodology (RFG and IRM), and any discrepancies were resolved with the intervention of a third reviewer (ILUV).

### 2.3. Selection Criteria and Data Extraction

Two independent reviewers (RFG and IRM) screened the titles, abstracts, and keywords of the retrieved studies following the Cochrane recommendations [26]. After selecting potentially eligible, relevant, peer-reviewed papers, full-text copies were reviewed and checked as to whether they met the inclusion criteria and to identify and record the reasons for excluding the ineligible studies. Disagreements were resolved by consensus, including a third reviewer (ILUV). Relevant data were extracted for each included study (RFG and IRM).

### 2.4. Methodological Quality and Risk of Bias Assessment

The PEDro scale was employed to assess the quality of the included trials because it is a reliable method for assessing RCT quality [27]. The total score for each study was stratified as follows: poor (<4 points), fair (4–5 points), good (6–8 points), and excellent (9–10 points) [28]. The risk of bias in each included study was assessed in accordance with the Cochrane recommendations, and a descriptive justification for the judgment was recorded following their guidelines [26]. In the “other bias” category, we clarified the specific criteria that could have affected the results. Detection bias was analyzed independently for objective physical variables and subjective patient-reported outcome measures.

Two independent trained assessors (RFG and IRM) examined the quality and risk of bias for the selected studies using the same methods, and disagreements were resolved by consensus or by consulting the third reviewer (ILUV). The inter-rater reliability was determined using the Kappa coefficient: >0.81–1.00 indicated excellent agreement between the assessors; 0.61–0.80 indicated good agreement; 0.41–0.60 indicated moderate agreement; and 0.21–0.40 indicated poor agreement [29].

### 2.5. Qualitative Analysis

The qualitative analysis was performed according to the Grading of Recommendations, Assessment, Development, and Evaluation [30].

### 2.6. Data Analysis

The statistical analysis was performed with RStudio 3.0 software, employing the “meta” and “esc” packages. All significance tests were conducted at a level of 5%. A meta-analysis was performed only when data for the analyzed variables were represented in at least 3 trials.

To increase the accuracy and thus the generalizability of our analyses, multiple trials from several studies (e.g., CoP path length in overhead squat test and CoP path length in single-leg squat test) were included in all analyses. Regarding the post-intervention period, the mean difference and standard deviation (SD) values were extracted for each outcome. When necessary, the mean scores and SDs were estimated from graphs. When the trial reported only standard errors, they were converted to SD in accordance with the Cochrane recommendations [26].

The summary statistics for all analyses were presented using forest plots. The raw mean difference (MD) was used as the overall effect size if studies used the same unit/tool of measurement. The standardized mean difference (SMD) was employed as the overall effect size if studies used different units/tools of measurement. A random-effects model was employed in all analyses to determine the overall effect size. The effect size of the statistical significance of the overall SMD was examined using Hedges’ g and interpreted as follows: trivial effect (g < 0.20); small effect (g = 0.20–0.49); moderate effect (g = 0.50–0.79); and large effect (g ≥ 0.80). The confidence interval around the pooled effect was calculated using the Knapp–Hartung adjustments [31].

The degree of heterogeneity among the studies was estimated using Cochran’s Q test and the inconsistency index (I^2^) [32]. Heterogeneity was considered when Cochran’s Q test was significant (*p* < 0.1) and/or the I^2^ was >50% [33]. To help with the clinical interpretation of the heterogeneity, the prediction interval (PI) based on the between-study variance tau-squared (τ^2^) were reported. The PI estimates the true intervention effect that can be expected in future settings [34]. As recommended for continuous outcomes [35], the restricted maximum likelihood estimator was used to calculate the between-study variance τ^2^.

The possible influence of the studies on the results obtained in the meta-analysis, as well as their robustness, were assessed with an exclusion sensitivity analysis. To detect publication bias, the funnel plots were visually assessed, with an asymmetric graph considered to indicate the presence of bias. The Luis Fury Kanamori (LFK) index was employed as a quantitative measure to detect publication bias [36]: no asymmetry (LFK within ±1); minor asymmetry (LFK exceeding ±1 but within ±2); and major asymmetry (LFK exceeding ±2). If there was significant asymmetry, a small-study effect method was applied to correct for publication bias using the Duval and Tweedie trim-and-fill method [37].

## 3. Results

### 3.1. Study Selection

**Figure 1:**
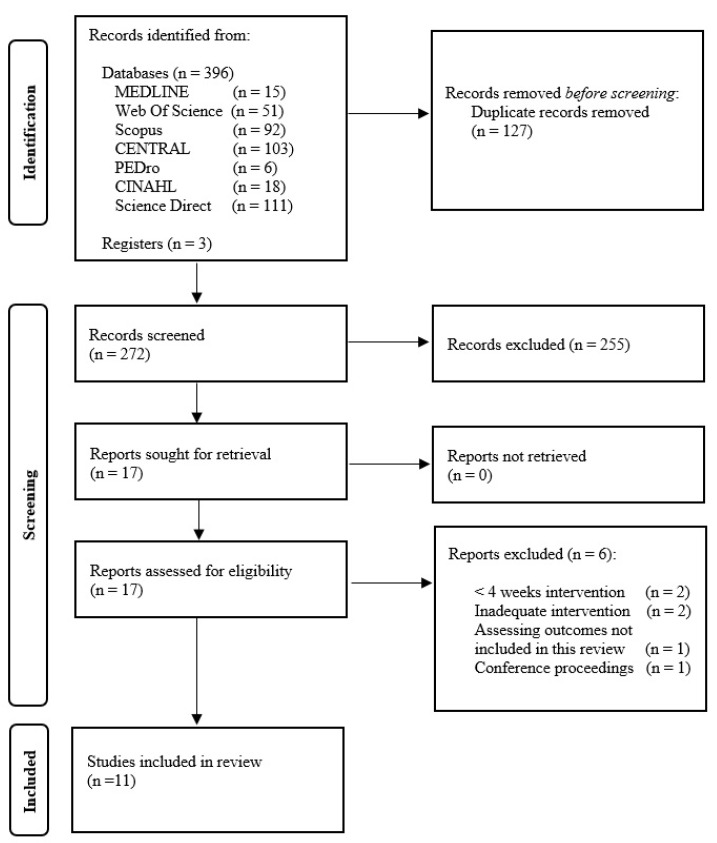
PRISMA flow diagram.

The search strategy yielded a total of 399 citations. After excluding articles not meeting the inclusion criteria, a total of 11 studies were selected for the final analysis. Figure 1 displays the flowchart of the search strategy.

### 3.2. Characteristics of the Included Studies

A total of 457 individuals with sub-acute and chronic LBP (mean age 29.89 years) were included in the studies (Table 1). Only the study by Janssens et al. [38] compared an RMT intervention with a sham RMT. A strength training program (frequently oriented toward the lower back) was performed in all groups, with the experimental group additionally performing an RMT program in 10 studies [14,15,16,39,40,41,42,43,44,45]. Seven studies assessed IMT alone, employing threshold loading devices [14,15,38,40,41,45], except for the Park at al. [44] study, which used the therapist’s hands to offer inspiration resistance. Four studies assessed combined training (IMT+EMT) with resistive loading devices [16,39,42,43]. The studies that used a threshold loading device applied either progressive [14,40,45] or stable [15,38,41] loads between 50% and 90% of MIP, with 60% being the most commonly selected MIP [20,40,43]; the training intensity ranged from “difficult” to “slightly difficult” (<14 in rated self-perceived exertion) [16,39,42,43]. The training programs were implemented between 4 [16,39,42] and 8 weeks [14,15,38,40,41,45] for 2 [15,41] and 7 [14,38,40,45] times per week (Table 1). None of the studies reported serious adverse events.

### 3.3. Methodological Quality and Risk of Bias of the Included Studies

The mean PEDro score of the included studies was 5.2 (range 4–6) (Appendix S2). The level of inter-evaluator agreement was high for inter-rater reliability (к = 0.895).

The risk of bias assessment of the included studies is summarized in Figure S1. In general, the risk of bias of the trials in the current meta-analysis was high. The highest risk of bias was found in the blinding of the outcome assessment and adequate stopping rules. However, all the included studies had an unclear risk of allocation concealment bias. The risk of bias in blinding the participants and assessors was judged low in all studies because the totality of the assessed studies adhered to strict protocols, a necessary requirement in exercise interventions to reduce the risk of differential behavior by the personnel delivering the intervention [46].

### 3.4. Functional Ability

**Figure 2:**
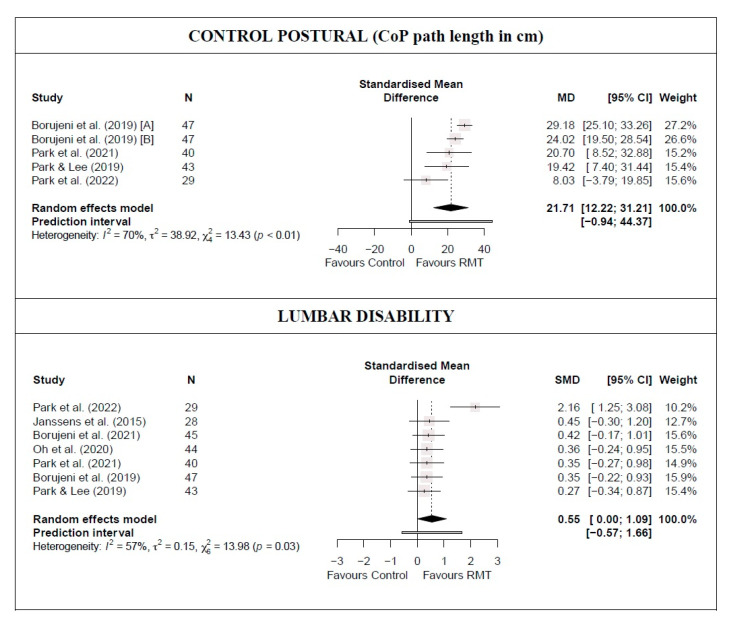
Synthesis forest plot for postural control and lumbar disability for respiratory muscle training versus control group [16,38,39,40,42,43,45].

There was low-quality evidence from four studies [39,42,43,45] that RMT produces a statistically significant increase in postural control (five trials; n = 159; MD = 21.71 cm [12.22; 31.21]), as well as a moderate and statistically significant decrease in lumbar disability (seven studies [16,38,39,40,43,45] [7 trials; n = 276]; SMD = 0.55 [0.001; 1.09]) compared with the control group (Figure 2 and Table 2). In both outcomes, heterogeneity was significant (I^2^ ≥ 57%) and the PI crossed zero; thus, future studies might find contradictory results. Based on the influence analyses, no single study significantly affected the overall MD in postural control. However, for the lumbar disability meta-analysis results, the study by Park et al. [43] was considered an outlier. The removal of this study still maintained the statistical significance of the estimated effect (small effect), reducing heterogeneity (I^2^ = 0%), and the PI did not cross zero (Figure 2 and Table 2), which will make the observed results more robust. Evidence of publication bias was detected in both outcomes (asymmetric funnel plot shape; major asymmetry for postural control [LFK index = −3.22]; and minor asymmetry for lumbar disability [LFK index = 1.67]; Figure S2). When the sensitivity analysis was adjusted for publication bias in both outcomes, there was no influence on the estimated effect because the trim-and-fill method considered that no studies should be added. Therefore, the initial results were maintained. 

### 3.5. Pain-Related Outcome Intensity and Pain-Related Fear-Avoidance Beliefs

**Figure 3:**
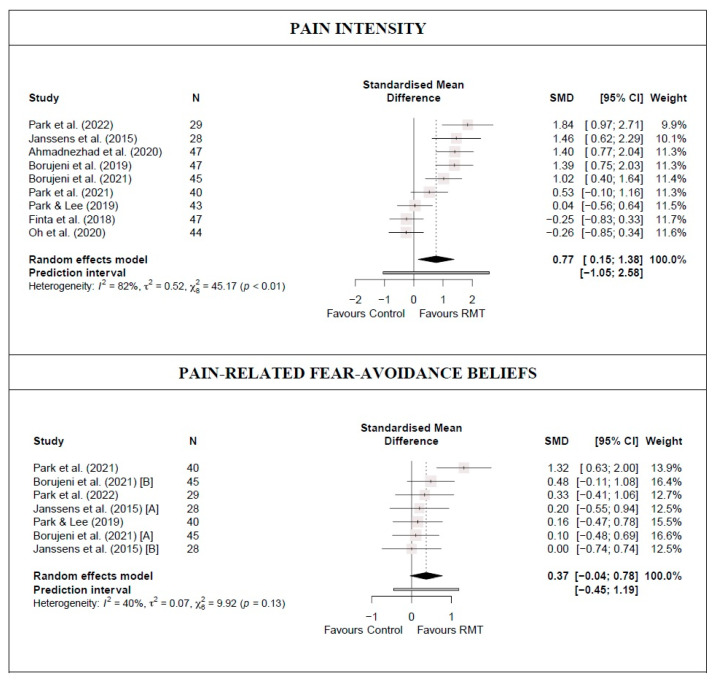
Synthesis forest plot for pain intensity and pain-related fear-avoidance beliefs for respiratory muscle training versus control group [14,15,16,38,39,40,42,43,45].

There was low-quality evidence from nine studies [14,15,16,38,39,40,42,43,45] (nine trials; n = 370) that RMT produces a moderate and statistically significant decrease in lumbar pain intensity compared with a control group (SMD = 0.77 [0.15; 1.38]; Figure 3 and Table 2). Heterogeneity was significant (I^2^ = 82%), and the PI crossed zero (−1.05; 2.58); thus, future studies might find contradictory results. Although no single study significantly affected the overall SMD, evidence of publication bias was detected (symmetric funnel plot shape; minor asymmetry [LFK index = 1.6]; Figure S3). When the sensitivity analysis was adjusted for publication bias, the initial results were maintained because the trim-and-fill method considered that no studies should be added.

There was moderate-quality evidence from five studies [38,39,40,42,43] (seven trials; n = 182) that RMT shows no statistically significant difference in reducing pain-related fear-avoidance beliefs compared with a control group (SMD = 0.37 [−0.04; 0.78)]; Figure 3 and Table 2). Heterogeneity was not significant (I^2^ = 40%). No single study significantly affected the overall SMD, and no evidence of publication bias was detected (symmetric funnel plot shape; no asymmetry [LFK index = 0.97]; Figure S3). 

### 3.6. Respiratory Function

**Figure 4:**
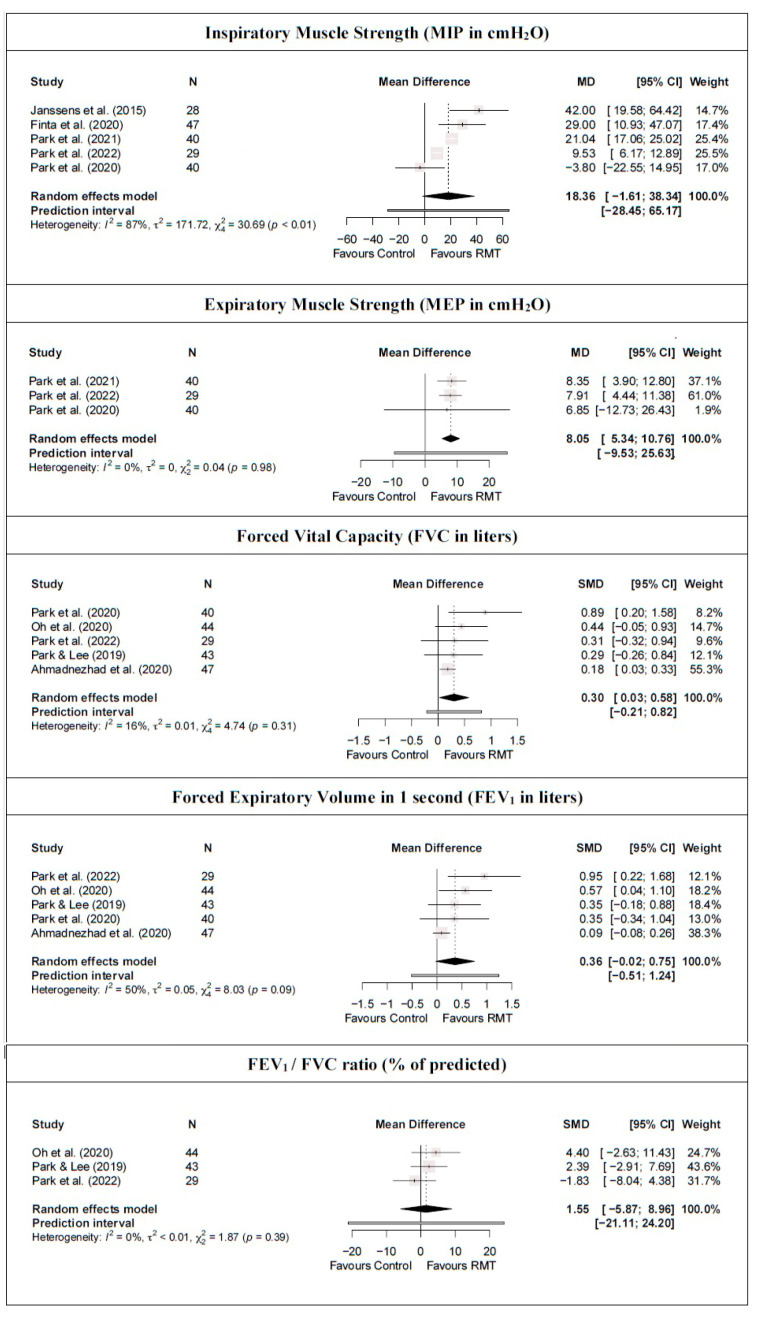
Synthesis forest plot for respiratory muscle strength and pulmonary function for respiratory muscle training versus control group [14,16,38,39,41,42,43,44].

For respiratory muscle strength, the meta-analysis results showed that RMT statistically significantly increases expiratory muscle strength (moderate-quality evidence from three studies [42,43,44] [three trials; n = 109]; MD = 8.05 cmH_2_O [5.34; 10.76]) but not inspiratory muscle strength (low-quality evidence from five studies [38,41,42,43,44] [five trials; n = 184]; MD = 18.36 cmH_2_O [−1.61; 38.34]) compared with a control group (Figure 4 and Table 2). Heterogeneity was significant for MIP (I^2^ = 87%) but not for MEP (I^2^ = 0%). In addition, the PI crossed zero (−9.53; 25.63) in the MEP meta-analysis results; thus, future studies might find contradictory results. Based on the influence analyses, no single study significantly affected the overall MD in MEP. However, the leave-one-out analysis for MIP suggested that by removing the Park et al. [44] study, RMT showed a statistically significant increase with respect to the control group. Evidence of publication bias was detected in both outcomes (asymmetric funnel plot shape; major asymmetry for MIP [LFK index = 3.83] and MEP [LFK index = −2.71]; Figure S4). When the sensitivity analysis was adjusted for publication bias, the initial results were maintained because the trim-and-fill method considered that no studies should be added.

For pulmonary function, there was moderate-quality evidence that RMT produces a statistically significant increase in FVC compared with a control group (FVC: five studies [14,16,39,43,44] [five trials; n = 203], MD = 0.30 L [0.03; 0.58]) but not in FEV1 and the FEV1/FVC ratio (FEV1: five studies [14,16,39,43,44] [five trials; n = 203], MD = 0.36 L [−0.02; 0.75]; FEV1/FVC: three studies [16,39,43] [three trials; n = 116], MD = 1.55% [−5.87; 8.96]; Figure 4 and Table 2). Heterogeneity was not significant for any pulmonary function outcomes (I^2^ ≤ 50%). No single study significantly affected the overall MD of the FEV1/FVC ratio meta-analysis results. However, the removal of the study by Oh et al. [16], Park et al. [43], or Park and Lee [39] implies an absence of statistically significant differences in FVC between the RMT and control groups. Similarly, removing the study by Ahmadnezhad et al. [14] shows that RMT produces a statistically significant increase in FEV1. In addition, evidence of publication bias was detected for FVC and FEV1 (symmetric funnel plot shape; major asymmetry [LFK index ≥ 4.65]; Figure S4). When the sensitivity analysis of these variables was adjusted for publication bias, the trim-and-fill method considered that three studies should be added. However, there was no influence on the estimated effect because the initial results were maintained.

## 4. Discussion

The main findings of the present systematic review and meta-analysis suggest that RMT could improve MEP and FVC, with a moderate quality of evidence, whereas a low quality of evidence suggests that RMT could improve postural control, lumbar disability, and pain intensity in individuals with sub-acute and chronic LBP. Given that the quality of the evidence supporting these findings is still low to moderate, more high-quality RCTs are required to confirm these trends.

Most individuals with LBP present altered motor control, impaired abdominal postural function [6], and lumbar segmental instability [5] partly due to the avoidance of movement. Lumbar stabilization is highly dependent on increased intra-abdominal pressure, which allows the unloading of the spine in weight-bearing and balance tasks without involving an excessive activation of the paraspinal muscles [38]. The main factor responsible for the increase in abdominal pressure is the co-contraction of the abdominal muscles [38]. This relationship between postural control and expiratory muscle strength was confirmed in this meta-analysis, in which both CoP path length and MEP improve after RMT in individuals with sub-acute and chronic LBP. RMT could induce hypertrophy [15] and improved the recruitment pattern [14] of the abdominal muscles in individuals with chronic LBP, leading to a positive impact on lumbar stability.

Lumbar disability was significantly decreased by RMT in individuals with sub-acute and chronic LBP. However, if one of the studies is excluded, the effect is not significant, which still reflects preliminary evidence. The most disabled individuals with chronic LBP were shown to demonstrate greater pain intensity, a longer duration of symptoms, more days off work, and poorer psychological well-being [47]. All these negative factors could be attenuated by RMT prescription and its subsequent disability reduction via greater abdominal stability and better neuromuscular function [48]. The Park et al. [43] study prescribed the longest intervention (5 weeks) and introduced significant heterogeneity in lumbar disability outcomes, which disappeared consistently when removed, confirming that RMT can improve lumbar disability in individuals with sub-acute and chronic LBP.

Although the stabilizing function of the diaphragm appears to be impaired in individuals with LBP [8,9], RMT has demonstrated positive effects in terms of diaphragm hypertrophy and activation [14,15,16]. The present review found no increases in inspiratory muscle strength after RMT in individuals with sub-acute and chronic LBP, but these results could have been negatively influenced by the validity of the RMT method employed in the Park et al. study [44] (therapist’s hands against the chest). The elimination of this study leads to significant improvements in MIP, which is consistent with prior literature [17,18] and is clinically meaningful [49].

The results of this review suggest that RMT, especially when combined with strength exercise, is more effective in reducing pain intensity than strength exercise alone in individuals with sub-acute and chronic LBP. This result is supported by a recent meta-analysis that confirmed (1) the analgesic effect of physical exercise and (2) that high training volumes and the addition of co-interventions reduce functional limitations in individuals with chronic LBP [50]. The gradual exposure of patients to movements and exercises that they had feared justifyies the overall reduction in fear-avoidance beliefs in all intervention groups [51], explaining the lack of significant results between groups found by this review. Notably, variables related to pain catastrophizing were not evaluated in the included studies. We suggest that future research incorporate these measures for a more comprehensive and precise assessment.

RMT appears to improve MEP, but not MIP, in individuals with LBP. However, the exclusion of Park et al.’s [44] study leads to significant improvements in MIP, which is in line with the previous literature on this population [14,15,16]. Therefore, RMT is able to modify the maximum respiratory pressures, which may influence the coordination and position of the diaphragm, found to be altered in individuals with LBP favoring fatigability and the persistence of the symptomology of chronic LBP [8,9]. RMT can increase the force-producing abilities of the diaphragm and abdominal muscles [52], resulting in a greater expansion of the lungs and chest walls, leading to increased chest wall compliance and increased FVC. However, it is unlikely that RMT modifies FEV1, given that it is directly dependent on the airway condition rather than on effort [53].

Overall, RMT appears to be a beneficial intervention for improving pain intensity, postural control, and lumbar disability, positively impacting on functionality and the quality of life of individuals with sub-acute and chronic LBP. RMT can be applied simultaneously with core-strengthening and stabilization exercises [15,16,39,41,42,43], which makes it an inexpensive, effective, and feasible therapeutic approach for this population. The simplicity of RMT allows independent performance by the patient, allowing the minimal control required by healthcare professionals, reducing the need for healthcare resources, and providing economic savings. Clinicians should consider incorporating RMT as a complementary intervention for individuals with sub-acute and chronic LBP. However, more high-quality randomized controlled trials are needed to strengthen the level of evidence supporting the effectiveness of RMT in this population, which is still low. Future studies should also investigate the optimal frequency, intensity, duration, and type of RMT, as well as the potential long-term effects and the cost-effectiveness of this intervention. Additionally, the mechanisms underlying the benefits of RMT in individuals with LBP should be further explored.

The current study presents several limitations. The low-to-moderate quality of evidence found in the variables analyzed suggests that the results should be interpreted with caution. The training periodicity and intensity varied widely, and the RMT devices were not homogeneous, with threshold and resistive devices and therapist’s hands being examined in the included studies. The heterogeneity in the protocols hinders obtaining clear conclusions on the effectiveness of RMT in individuals with sub-acute and chronic LBP. The inclusion of individuals with sub-acute and chronic low back pain limits the generalizability of the findings to those with acute pain or other types of low back pain. Due to the limited number of studies included, it was impossible to perform subanalyses to address this clinical heterogeneity. The lack of long-term follow-up data in the included studies limits the ability to draw conclusions about the sustained effectiveness of RMT for sub-acute and chronic LBP. Future reviews should address these issues and more high-quality RCTs are required in different LBP populations.

## 5. Conclusions

The current systematic review and meta-analysis indicates that RMT could improve expiratory muscle strength and FVC in individuals with sub-acute and chronic LBP, with moderate-quality evidence. Low-quality evidence suggests that RMT could enhance postural control, reduce lumbar disability, and decrease pain intensity in the same population. The quality of the evidence supporting these findings presents room for improvement, and the heterogeneity of training protocols is still high. More high-quality RCTs are required to confirm the effects of RMT in individuals with sub-acute and chronic LBP.

## Figures and Tables

**Table 1:** Methodological characteristics and results of the included studies.

Study	Participants	Intervention Groups	Training Protocol	Outcomes	Key Results
Ahmad-nezhad et al., 2020 [14] RCT	*N* = 47; 24 females (51%) LBP > 6 months Age: 21.89 ± 1.85 years	IMT+RT (*N* = 23) RT (*N* = 24)	IMT: 30 breaths twice a day; 7 days/week; 8 weeks. 50 to 90% MIP. Threshold loading device RT: Weightlifting and powerlifting training	Pain intensity: VAS Respiratory function: FVC and FEV_1_	Pain intensity: The IMT+RT group showed significant reductions for pain compared with the RT group at 8 weeks. Respiratory function: The IMT+RT group showed significant improvements for FVC and FEV_1_ compared with the RT group at 8 weeks.
Borujeni et al., 2019 [45] RCT	*N* = 47; 25 females (53%) LBP > 6 months Age: 21.63 ± 1.69 years	IMT+RT (*N* = 24) RT (*N* = 23)	IMT: 30 breaths twice a day; 7 days/week; 8 weeks. 50 to 90% MIP. Threshold loading device RT: Weightlifting and powerlifting training	Postural control: CoP path length Lumbar disability: ADI Pain intensity: VAS	Postural control: The IMT+RT group showed significant improvements for CoP path length compared with the RT group at 8 weeks. Lumbar disability: No differences were found in ADI between the groups at 8 weeks. Pain intensity: The IMT+RT group showed significant reductions in pain compared with the RT group at 8 weeks.
Borujeni et al., 2021 [40] RCT	*N* = 45; 23 females (51%) LBP > 3 months Age: 21.56 ± 2.16 years	IMT+RT (*N* = 23) RT (*N* = 22)	IMT: 30 breaths twice a day; 7 days/week; 8 weeks. 50 to 85% MIP. Threshold loading device RT: Weightlifting and powerlifting training	Lumbar disability: ADI Pain intensity: VAS Pain-related fear-avoidance beliefs: TSK and FABQ	Lumbar disability: No differences were found in ADI between the groups at 8 weeks. Pain intensity: The IMT+RT group showed significant reductions in pain compared with the RT group at 8 weeks. Pain-related fear-avoidance beliefs: No differences were found in the TSK or FABQ between the groups at 8 weeks.
Finta et al., 2018 [15] RCT	*N* = 47; 33 females (70%) LBP > 3 months Age: 21.87 ± 4.94 years	IMT+LSE (*N* = 26) LSE (*N* = 21)	IMT: 30 breaths twice a day; 2 days/week; 8 weeks. Also applied during conventional training 60% MIP. Threshold loading device LSE: 60 min: Balance, trunk muscles strengthening, mobilizing, and stretching. 2 days/week; 8 weeks	Pain intensity: VAS	Pain intensity: Both groups showed significant reductions in pain VAS at 8 weeks.
Finta et al., 2020 [41] RCT	*N* = 47; 33 females (70%) LBP > 3 months Age: 21.87 ± 4.94 years	IMT+LSE (*N* = 26) LSE (*N* = 21)	IMT: 30 breaths twice a day; 2 days/week; 8 weeks. Also applied during conventional training. 60% MIP. Threshold loading device LSE: 60 min: Balance, trunk muscles strengthening, mobilizing, and stretching. 2 days/week; 8 weeks	Respiratory function: MIP	Respiratory function: The IMT+LSE group showed a significant increases in MIP compared with the LSE group at 8 weeks.
Janssens et al., 2015 [38] RCT	*N* = 28, females 18 (64%) LBP > 6 months Age: 32.5 ± 7.93 years	IMT (*N* = 14) Sham IMT (*N* = 14)	IMT: 30 breaths twice a day; 7 days/week; 8 weeks. 60% MIP. Threshold loading device Sham IMT Same protocol applying an intensity of 10% MIP. Threshold loading device.	Lumbar disability: ODI Pain intensity: NRS Pain-related fear-avoidance beliefs: TSK and FABQ Respiratory function: MIP	Lumbar disability: No differences were found in ODI between the groups at 8 weeks. Pain intensity: The IMT+LSE group showed significant reductions in pain compared with the LSE group at 8 weeks. Pain-related fear-avoidance beliefs: No differences were found in the TSK or FABQ between the groups at 8 weeks. Respiratory function: The IMT+LSE group showed significant increases in MIP compared with the LSE group at 8 weeks.
Oh et al., 2020 [16] RCT	*N* = 44; females 44 (100%) LBP > 6 weeks Age: 45.30 ± 2.68 years	RMT+LSE (*N* = 22) LSE (*N* = 22)	RMT: Applied during LSE 3 days/week; 4 weeks. RPE below 14. Resistive loading device. LSE: 50 min: 3–5 sets of five core-strengthening exercises. 20 s each set; 1 min rest interval. RPE below 14. 3 days/week; 4 weeks	Lumbar disability: ODI Pain intensity: VAS Respiratory function: FVC, FEV_1_, and FEV_1_/FVC	Lumbar disability: The IMT+LSE group showed significant improvements in ODI compared with the LSE group at 4 weeks. Pain intensity: No differences were found in pain between the groups at 4 weeks. Respiratory function: The IMT+LSE group showed significant improvements only in FVC and FEV_1_ compared with the LSE group at 4 weeks.
Park and Lee. 2019 [39] RCT	*N* = 43; females 19 (44%) LBP > 6 weeks Age: 30.79 ± 5.50 years	RMT+LSE (*N* = 20) LSE (*N* = 23)	RMT: Applied during LSE 3. days/week; 4 weeks. RPE below 14. Resistive loading device LSE: 40 min: three sets × five reps (20 s each rep) of five core-strengthening exercises. 1 min rest interval. 3 days/week; 4 weeks	Postural control: CoP path length Lumbar disability: ODI Pain intensity: NRS Pain-related fear-avoidance beliefs: FABQ Respiratory function: FVC, FEV_1_, and FEV_1_/FVC	Postural control: No differences were found in CoP path length between the groups at 4 weeks. Lumbar disability: No differences were found in ODI between the groups at 4 weeks. Pain intensity: No differences were found in pain between the groups at 4 weeks. Pain-related fear-avoidance beliefs: The IMT+LSE group showed significant improvements only in the FABQ of physical activity compared with the LSE group at 4 weeks. Respiratory function: The IMT+LSE group showed significant improvements only in FVC compared with the LSE group at 4 weeks.
Park et al., 2020 [44] RCT	*N* = 40; females not reported LBP > 3 months Age: 39.85 ± 9.19 years	IMT+LSE (*N* = 20) LSE (*N* = 20)	IMT: Inspiratory resistance was applied through therapist’s hands for 10 min, 5 days/week; 6 weeks. Intensity not reported LSE: 20 min: Re-education, static and dynamic stability exercises. 3 days/week; 4 weeks	Respiratory function: MIP, MEP, FVC, and FEV_1_	Respiratory function: Both study groups showed significant improvements in MIP, MEP FVC, and FEV_1_ at 6 weeks.
Park et al., 2021 [42] RCT	*N* = 40; females 40 (100%) CLBP > 6 weeks Age: 46.89 ± 6.49 years	RMT+LSE (*N* = 20) LSE (*N* = 20)	RMT: Applied during LSE. 3 days/week; 4 weeks. RPE below 14. Resistive loading device LSE: 60 min: five sets × five reps (10 s each rep) of six core-strengthening exercises. 20 s rest interval. 3 days/week; 4 weeks	Postural Control: CoP path length Lumbar disability: ODI Pain intensity: NRS Pain-related fear-avoidance beliefs: FABQ Respiratory function: MIP and MEP	Postural Control: The IMT+LSE group showed significant improvements in CoP path length compared with the LSE group at 4 weeks. Lumbar disability: The IMT+LSE group showed significant improvements in ODI compared with the LSE group at 4 weeks. Pain intensity: No differences were found in pain NRS between the groups at 4 weeks. Pain-related fear-avoidance beliefs: The IMT+LSE group showed significant improvements in the FABQ compared with the LSE group at 4 weeks. Respiratory function: The IMT+LSE group showed significant improvements in MIP and MEP compared with the LSE group at 4 weeks.
Park et al., 2022 [43] RCT	*N* = 29; females 13 (45%) CLBP > 6 weeks Age: 30.67 ± 6.00 years	RMT+LSE (*N* = 14) LSE (*N* = 15)	RMT: Applied during LSE. 3 days/week; 5 weeks. RPE 13–14. Resistive loading device LSE: 60 min: five sets × five reps (10 s each rep) of six core-strengthening exercises. 20 s rest interval. 3 days/week; 5 weeks.	Postural Control: CoP path length Lumbar disability: RMQD Pain intensity: VAS Pain-related fear-avoidance beliefs: FABQ Respiratory function: MIP, MEP, FVC, FEV_1_, andFEV_1_/FVC	Postural Control: No differences were found between the groups. Lumbar disability: The IMT+LSE group showed significant improvements in the RMDQ compared with the LSE group at 5 weeks. Pain intensity: The IMT+LSE group showed significant improvements in pain compared with the LSE group at 5 weeks. Pain-related fear-avoidance beliefs: No differences were found in the FABQ between the groups at 5 weeks. Respiratory function: The IMT+LSE group showed significant improvements in all respiratory function outcomes compared with the LSE group at 4 weeks.

ADI: athletes disability index; CLBP: chronic low back pain; CoP: centre of pressure; FABQ: fear-avoidance beliefs questionnaire; FEV1: forced expiratory volume at the first second; FVC: forced vital capacity; IMT: inspiratory muscle training; LSE: lumbar stabilization exercise; MEP: maximal expiratory pressure; Min: minute; MIP: maximal inspiratory pressure; NRS: numerical rating scale; ODI: oswestry disability index; RCT: randomized controlled trial; Rep: repetition; RMQD: roland-morris low back pain and disability questionnaire; RMT: respiratory muscle training; RPE: rate of perceived exertion; RT: resistance training; SD: standard deviation; TSK: tampa scale for kinesiophobia; VAS: visual analogue scale.

**Table 2:** GRADE evidence profile for the effects of respiratory muscle training.

Outcome Comparison; Number of Studies (Trials); Sample Size	Risk of Bias	Inconsistency	Indirectness of Evidence	Imprecision	Publication Bias	SMD (95% CI) or MD (95% CI)	Certainty of Evidence
Functional ability							
Postural control (center of pressure in cm)							LOW
RMT vs. control (overall effect); four studies (five trials); n = 159	Not serious	Serious ^a^	Not serious	Serious ^b^	Not serious	21.71 cm (12.22 to 31.21) *	⨁⨁◯◯
Lumbar disability (SMD)							LOW
RMT vs. control (overall effect); seven studies (seven trials); n = 276	Not serious	Serious ^a^	Not serious	Serious ^b^	Not serious	0.55 (0.001 to 1.09) *	⨁⨁◯◯
Pain-related outcomes							
Pain intensity (SMD)							LOW
RMT vs. control (overall effect); nine studies (nine trials); n = 370	Not serious	Serious ^a^	Not serious	Serious ^b^	Not serious	0.77 (0.15 to 1.38) *	⨁⨁◯◯
Pain-related fear-avoidance beliefs (SMD)							MODERATE
RMT vs. control (overall effect); five studies (seven trials); n = 182	Not serious	Not serious	Not serious	Serious ^b^	Not serious	0.37 (−0.04 to 0.78)	⨁⨁⨁◯
Respiratory muscle function							
Inspiratory muscle strength (MIP in cmH_2_O)							LOW
RMT vs. control (overall effect); five studies (five trials); n = 184	Not serious	Serious ^a^	Not serious	Serious ^b^	Not serious	18.36 cmH_2_O (−1.61 to 38.34)	⨁⨁◯◯
Expiratory muscle strength (MEP in cmH_2_O)							MODERATE
RMT vs. control (overall effect); three studies (three trials); n = 109	Not serious	Not serious	Not serious	Serious ^b^	Not serious	8.05 cmH_2_O (5.34 to 10.76) *	⨁⨁⨁◯
Respiratory function							
Forced vital capacity (FVC in liters)							MODERATE
RMT vs. control (overall effect); five studies (five trials); n = 203	Not serious	Not serious	Not serious	Serious ^b^	Not serious	0.30 L (0.03 to 0.58) *	⨁⨁⨁◯
Forced expiratory volume at 1º s (FEV_1_ in liters)							MODERATE
RMT vs. control (overall effect); five studies (five trials); n = 203	Not serious	Not serious	Not serious	Serious ^b^	Not serious	0.36 L (−0.02 to 0.75)	⨁⨁⨁◯
Ratio FEV_1_/FVC (% of predicted)							MODERATE
RMT vs. control (overall effect); three studies (three trials); n = 116	Not serious	Not serious	Not serious	Serious ^b^	Not serious	1.55% (−5.87 to 8.96)	⨁⨁⨁◯

* Statistically significant differences; ^a^ I^2^ > 50%; ^b^ sample size less than 400 patients. MD, mean difference; MIP, maximal inspiratory pressure; MEP, maximal expiratory pressure; RMT, respiratory muscle training; SMD, standardized mean difference.

## Data Availability

No new data were created.

## Supplementary Materials

The following supporting information can be downloaded at: https://www.mdpi.com/article/10.3390/jcm13113053/s1, Appendix S1: Full search strategy; Appendix S2: PEDro scores for included studies (n = 11); Figure S1: Risk of bias summary and graph; Figure S2. Sensitivity and publication bias funnel plots for the comparison in postural control and lumbar disability; Figure S3: Sensitivity and publication bias funnel plots for the comparison in pain intensity and pain-related fear-avoidance beliefs; Figure S4: Sensitivity and publication bias funnel plots for the comparison in inspiratory and expiratory muscle strength, forced vital capacity, forced expiratory volume in 1 s, and ratio FEV1/FVC.
