# Supplementary material for: Effects of Respiratory Muscle Training on Functional Ability, Pain-Related Outcomes, and Respiratory Function in Individuals with Low Back Pain: Systematic Review and Meta-Analysis
Source: J Clin Med. 2024 May 23;13(11):3053. doi: 10.3390/jcm13113053 (PMC11172635; doi:10.3390/jcm13113053)
Supplement: Supplementary file 1 [file jcm-13-03053-s001.zip › Appendix S1. search strategy BPain+RMT.pdf]

Appendix S1. Full search strategy

MEDLINE

1. “respiratory muscle strength training” [Title/Abstract] OR “respiratory muscle training” [Title/Abstract] OR “respiratory muscle exercise” [Title/Abstract] OR “RMT” [Title/Abstract] OR “respiratory resistance” [Title/Abstract] OR “diaphragm training” [Title/Abstract]
2. “inspiratory muscle strength training” [Title/Abstract] OR “inspiratory muscle training” [Title/Abstract] OR “inspiratory muscle exercise” [Title/Abstract] OR “IMT” [Title/Abstract]
3. “expiratory muscle strength training” [Title/Abstract] OR “expiratory muscle training” [Title/Abstract] OR “expiratory muscle exercise” [Title/Abstract] OR “EMT” [Title/Abstract]
4. “breathing exercises” [Title/Abstract]
5. #1 OR #2 OR #3 OR #4
6. "low back pain" [Title/Abstract] OR "lumbar pain" [Title/Abstract] OR "chronic low back pain" [Title/Abstract] OR "non specific lumbar pain" [Title/Abstract] OR "non specific low back pain" [Title/Abstract] OR “lumbago” [Title/Abstract] OR “low back ache” [Title/Abstract] OR “mechanical low back pain” [Title/Abstract]
7. "back injury" [Title/Abstract] OR "disc degeneration" [Title/Abstract] OR “backache” [Title/Abstract] OR “back ache” [Title/Abstract] OR "back pain" [Title/Abstract]
8. #6 OR #7 OR #8 OR #9
9. ((clinical[Title/Abstract] AND trial[Title/Abstract]) OR clinical trials as topic[MeSH Terms] OR "clinical trial" [Publication Type] OR

random*[Title/Abstract] OR "random allocation" [MeSH Terms] OR therapeutic use[MeSH Subheading])

10. #5 AND #10 AND #11

WEB OF SCIENCE

1. Topic: “respiratory muscle strength training” OR “respiratory muscle training” OR “respiratory muscle exercise” OR “respiratory resistance” OR “diaphragm training” OR “IMT” OR “EMT” OR “RMT” OR “inspiratory muscle strength training” OR “inspiratory muscle training” OR “inspiratory muscle exercise” OR “expiratory muscle strength training” OR “expiratory muscle training” OR “expiratory muscle exercise” OR “breathing exercises”
2. Topic: "low back pain" OR "lumbar pain" OR "chronic low back pain" OR "non specific lumbar pain" OR "non specific low back pain" OR “lumbago” OR “low back ache” OR “mechanical low back pain” OR "back injury" OR "disc degeneration" OR “backache” OR “back ache” OR "back pain"
3. Topic: "randomized controlled trial" OR "controlled clinical trial" OR "random" OR "randomly" OR "trial"
4. #1 AND #2 AND #3

Scopus

1. [Article title, abstract, keywords]: “respiratory muscle strength training” OR “respiratory muscle training” OR “respiratory muscle exercise” OR “respiratory resistance” OR “diaphragm training” OR “IMT” OR “EMT” OR “RMT” OR “inspiratory muscle strength training” OR “inspiratory muscle training” OR “inspiratory muscle exercise” OR “expiratory muscle strength training” OR

“expiratory muscle training” OR “expiratory muscle exercise” OR “breathing exercises”

2. [Article title, abstract, keywords]: "low back pain" OR "lumbar pain" OR "chronic low back pain" OR "non specific lumbar pain" OR "non specific low back pain" OR “lumbago” OR “low back ache” OR “mechanical low back pain” OR "back injury" OR "disc degeneration" OR “backache” OR “back ache” OR "back pain"
3. [Article title, abstract, keywords]: "randomized controlled trial" OR "controlled clinical trial" OR "random" OR "randomly" OR "trial"
4. #1 AND #2 AND #3

Cochrane Controlled Register of Trials (CENTRAL)

1. [Title, abstract, keyword]: “respiratory muscle strength training” OR “respiratory muscle training” OR “respiratory muscle exercise” OR “respiratory resistance” OR “diaphragm training” OR “IMT” OR “EMT” OR “RMT” OR “inspiratory muscle strength training” OR “inspiratory muscle training” OR “inspiratory muscle exercise” OR “expiratory muscle strength training” OR “expiratory muscle training” OR “expiratory muscle exercise” OR “breathing exercises”
2. [Title, abstract, keyword]: "low back pain" OR "lumbar pain" OR "chronic low back pain" OR "non specific lumbar pain" OR "non specific low back pain" OR “lumbago” OR “low back ache” OR “mechanical low back pain” OR "back injury" OR "disc degeneration" OR “backache” OR “back ache” OR "back pain"
3. #1 AND #2

PEDro

Abstract & Title: respiratory muscle training and back pain

Therapy: no selection

Problem: pain

Body part: no selection

Subdiscipline: no selection

Topic: No selection

Title only: no selection

Method: Clinical Trial

Match all search terms (AND): Yes

CINAHL

Search mode: Boolean/Phrase

Apply equivalent subjects: Yes

Journal Subset: All

Language: All

Sex: All

Age groups: All

Clinical Queries: All

Geographic Subste: All

Publication type: All

Special Interest: All

3. No Field: “respiratory muscle strength training” OR “respiratory muscle training” OR “respiratory muscle exercise” OR “respiratory resistance” OR “diaphragm training” OR “IMT” OR “EMT” OR “RMT” OR “inspiratory

muscle strength training” OR “inspiratory muscle training” OR “inspiratory muscle exercise” OR “expiratory muscle strength training” OR “expiratory muscle training” OR “expiratory muscle exercise” OR “breathing exercises”

4. No Field: "low back pain" OR "lumbar pain" OR "chronic low back pain" OR "non specific lumbar pain" OR "non specific low back pain" OR “lumbago” OR “low back ache” OR “mechanical low back pain” OR "back injury" OR "disc degeneration" OR “backache” OR “back ache” OR "back pain"
5. No Field: "randomized controlled trial" OR "controlled clinical trial" OR "random" OR "randomly" OR "trial"
6. #1 AND #2 AND #3

Science Direct

Find articles with these terms: ("chronic low back pain" OR “lumbar pain" OR “lumbago” OR “low back ache” OR "back pain") AND ("respiratory muscle training" OR "inspiratory muscle training" OR "expiratory muscle training")
