# Supplementary material for: Effects of Respiratory Muscle Training on Functional Ability, Pain-Related Outcomes, and Respiratory Function in Individuals with Low Back Pain: Systematic Review and Meta-Analysis
Source: J Clin Med. 2024 May 23;13(11):3053. doi: 10.3390/jcm13113053 (PMC11172635; doi:10.3390/jcm13113053)
Supplement: Supplementary file 1 [file jcm-13-03053-s001.zip › Appendix S2. PEDro scores.pdf]

Appendix S2. PEDro scores for included studies (n = 11).

Study	Random allocation	Concealed allocation	Groups similar at baseline	Participant blinding	Therapist blinding	Assessor blinding	< 15% dropouts	Intention -to-treat analysis	Between-group difference reported	Point estimate and variability reported	TOTAL
Ahmadnezhad 2020 [14]	Y	N	Y	N	N	Y	Y	N	Y	Y	**6**
Borujeni & Yalfani 2019 [46]	Y	N	Y	N	N	Y	Y	N	Y	Y	**6**
Borujeni & Yalfani 2021 [41]	Y	N	Y	N	N	Y	Y	N	Y	Y	**6**
Finta 2018 [15]	Y	N	Y	N	N	N	Y	N	N	Y	**4**
Finta 2020 [42]	Y	N	Y	N	N	N	Y	N	Y	Y	**5**
Janssens 2015 [40]	Y	N	Y	Y	N	N	Y	N	Y	Y	**6**
Oh 2020 [16]	Y	N	Y	N	N	N	Y	N	Y	Y	**5**
Park & Lee 2019 [39]	Y	N	Y	N	N	N	Y	N	Y	Y	**5**
Park 2020 [45]	Y	N	Y	N	N	N	N	N	Y	Y	**4**
Park 2021[43]	Y	N	Y	N	N	N	Y	N	Y	Y	**5**
Park 2022 [44]	Y	N	Y	N	N	N	Y	N	Y	Y	**5**
N = No, Y = Yes									Mean		**5.2/10**
