# Supplementary material for: Effects of Respiratory Muscle Training on Functional Ability, Pain-Related Outcomes, and Respiratory Function in Individuals with Low Back Pain: Systematic Review and Meta-Analysis
Source: J Clin Med. 2024 May 23;13(11):3053. doi: 10.3390/jcm13113053 (PMC11172635; doi:10.3390/jcm13113053)
Supplement: Supplementary file 1 [file jcm-13-03053-s001.zip › Figure S1. Risk of bias summary and graph copia.pdf]

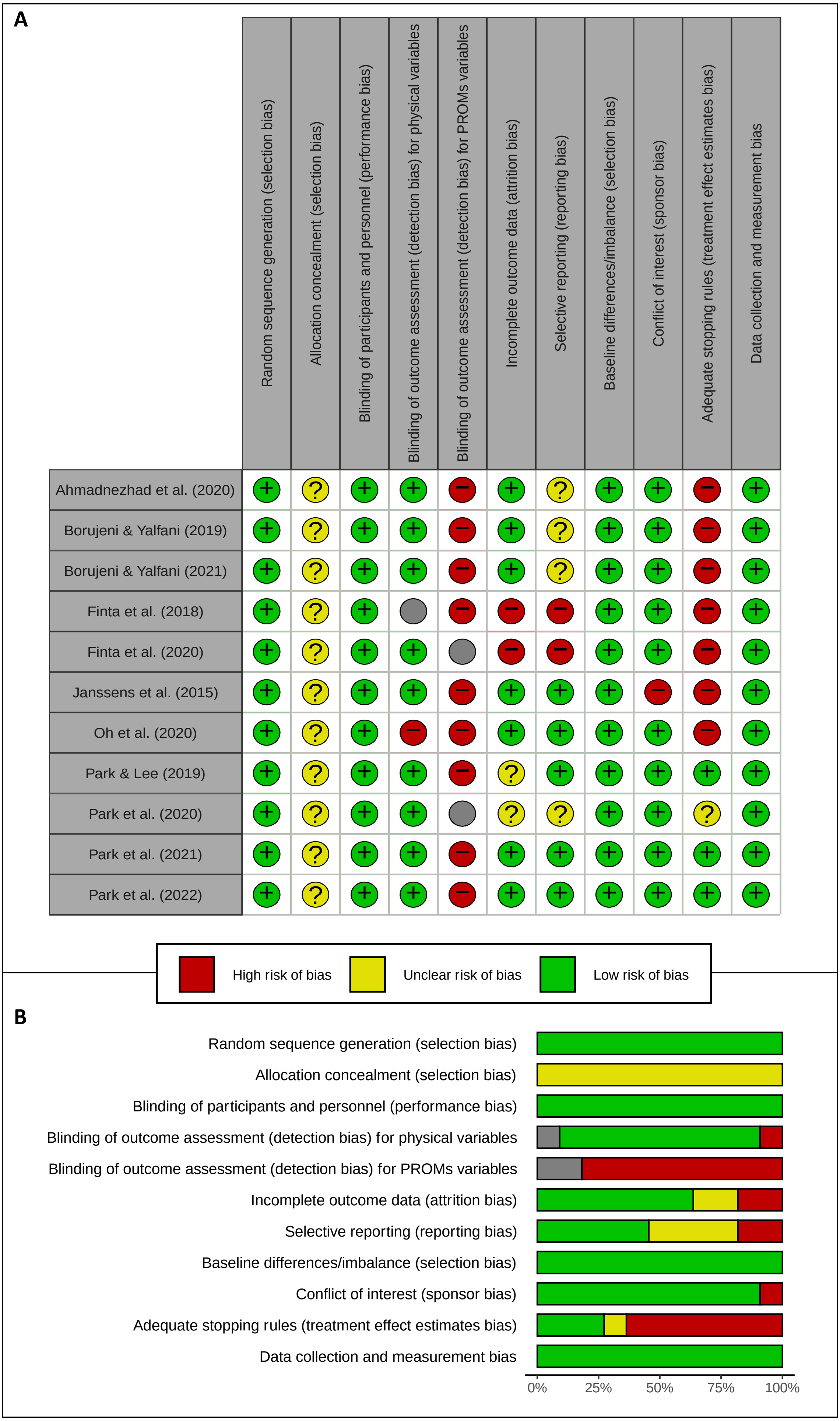

A. Risk of bias summary: review authors' judgements about each Risk of bias item for each included study.

B. Risk of bias graph: review authors' judgements about each Risk of bias item presented as percentages across all included studies.
