# Supplementary material for: Effects of Respiratory Muscle Training on Functional Ability, Pain-Related Outcomes, and Respiratory Function in Individuals with Low Back Pain: Systematic Review and Meta-Analysis
Source: J Clin Med. 2024 May 23;13(11):3053. doi: 10.3390/jcm13113053 (PMC11172635; doi:10.3390/jcm13113053)

POSTURAL CONTROL (CoP path length)

A. Leave-one-out sensitivity analysis

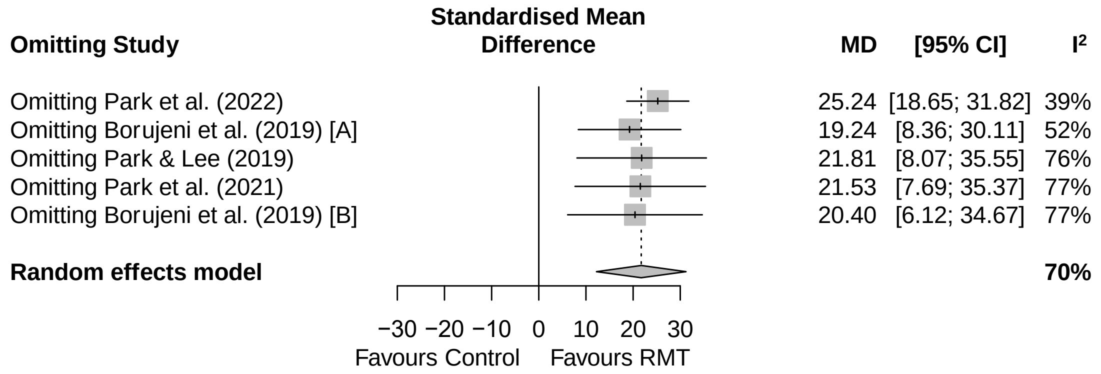

B. Publication bias

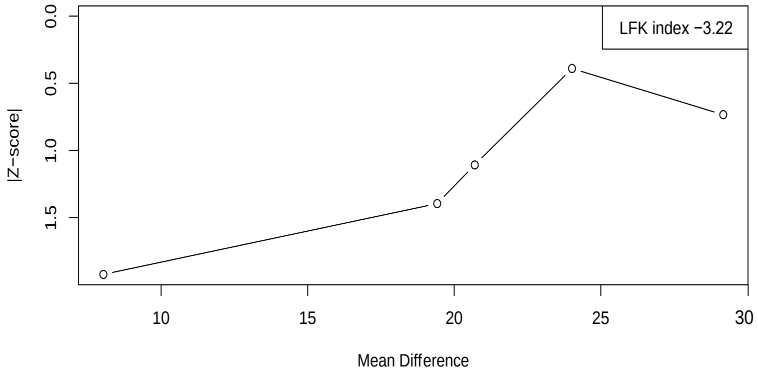

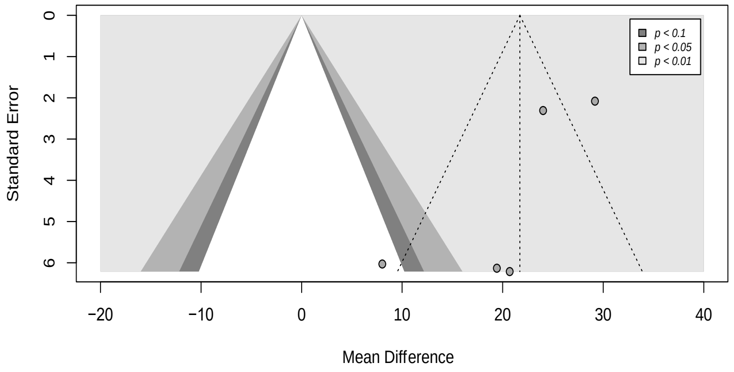

LUMBAR DISABILITY

A. Leave-one-out sensitivity analysis

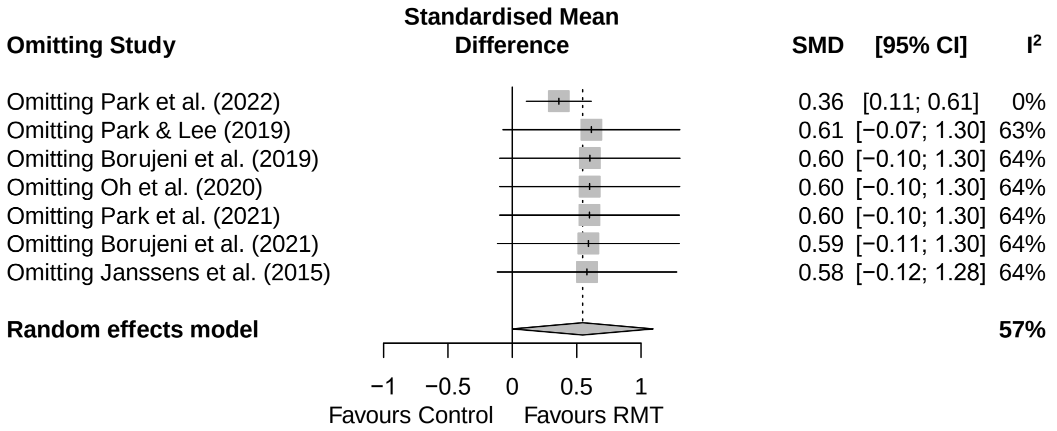

B. Publication bias

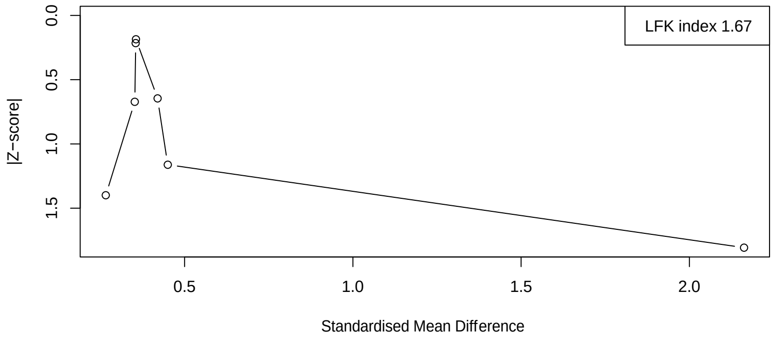

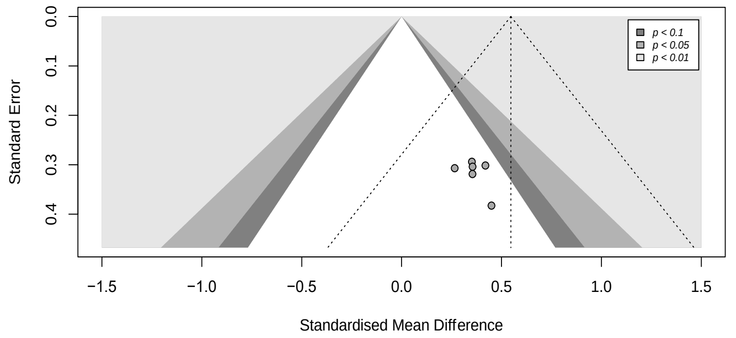

Supplement: Supplementary file 1 [file jcm-13-03053-s001.zip › Figure S2. Sensitivity Functional Ability.pdf]
