# Supplementary material for: Effects of Respiratory Muscle Training on Functional Ability, Pain-Related Outcomes, and Respiratory Function in Individuals with Low Back Pain: Systematic Review and Meta-Analysis
Source: J Clin Med. 2024 May 23;13(11):3053. doi: 10.3390/jcm13113053 (PMC11172635; doi:10.3390/jcm13113053)

PAIN INTENSITY

A. Leave-one-out sensitivity analysis

Omitting Study	SMD	[95% CI]	I^2
Omitting Finta et al. (2018)	0.90	[0.27; 1.52]	80%
Omitting Oh et al. (2020)	0.90	[0.27; 1.52]	80%
Omitting Park et al. (2022)	0.65	[0.02; 1.27]	81%
Omitting Ahmadnezhad et al. (2020)	0.69	[0.00; 1.37]	82%
Omitting Borujeni et al. (2019)	0.69	[0.01; 1.37]	82%
Omitting Park & Lee (2019)	0.86	[0.19; 1.53]	83%
Omitting Janssens et al. (2015)	0.69	[0.01; 1.37]	83%
Omitting Borujeni et al. (2021)	0.74	[0.03; 1.45]	84%
Omitting Park et al. (2021)	0.80	[0.09; 1.51]	84%
**Random effects model**			**82%**

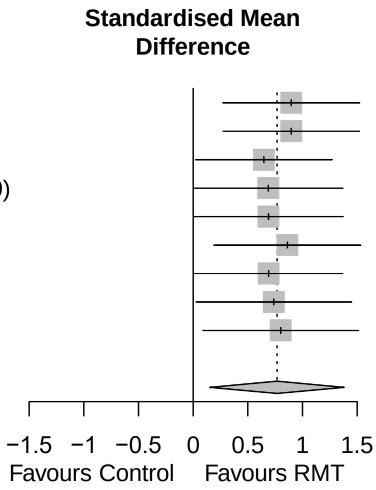

B. Publication bias

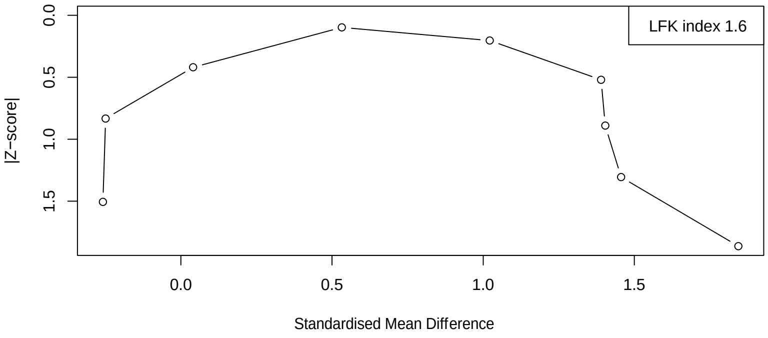

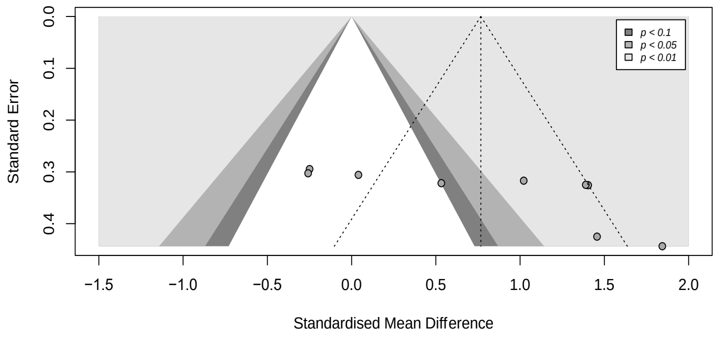

PAIN-RELATED FEAR-AVOIDANCE BELIEFS

A. Leave-one-out sensitivity analysis

Omitting Study	SMD	[95% CI]	I^2
Omitting Park et al. (2021)	0.22	[−0.05; 0.49]	0%
Omitting Janssens et al. (2015) [B]	0.42	[−0.05; 0.89]	44%
Omitting Borujeni et al. (2021) [A]	0.42	[−0.07; 0.91]	44%
Omitting Park & Lee (2019)	0.41	[−0.09; 0.91]	47%
Omitting Janssens et al. (2015) [A]	0.39	[−0.10; 0.89]	48%
Omitting Borujeni et al. (2021) [B]	0.35	[−0.16; 0.86]	49%
Omitting Park et al. (2022)	0.38	[−0.13; 0.88]	50%
**Random effects model**			**40%**

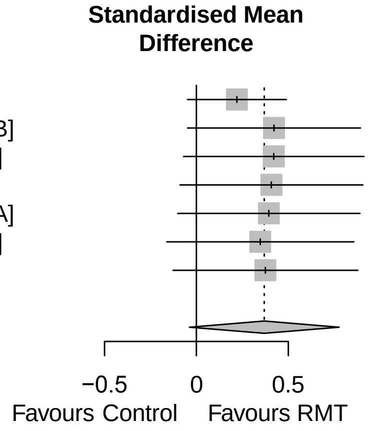

B. Publication bias

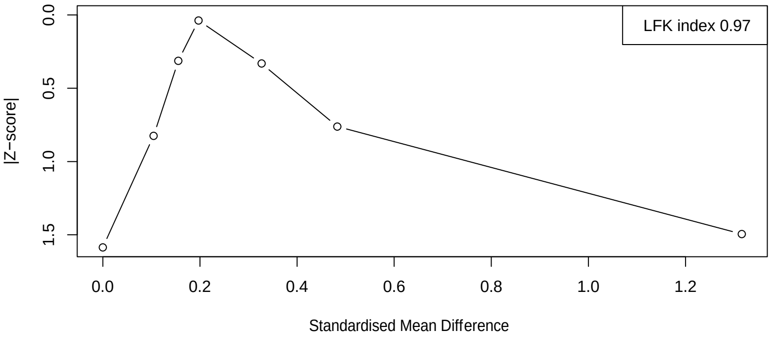

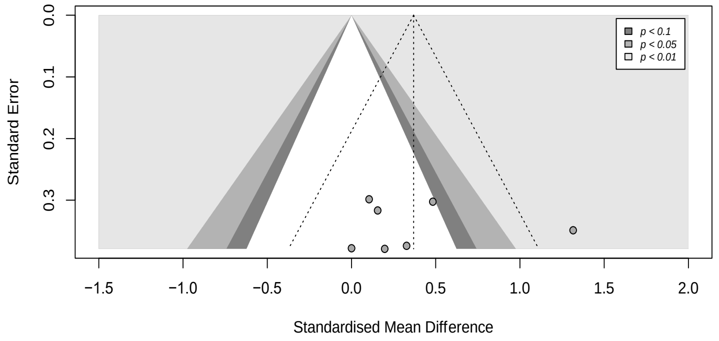

Supplement: Supplementary file 1 [file jcm-13-03053-s001.zip › Figure S3. Sensitivity pain outcomes.pdf]
