# Supplementary material for: Effects of Respiratory Muscle Training on Functional Ability, Pain-Related Outcomes, and Respiratory Function in Individuals with Low Back Pain: Systematic Review and Meta-Analysis
Source: J Clin Med. 2024 May 23;13(11):3053. doi: 10.3390/jcm13113053 (PMC11172635; doi:10.3390/jcm13113053)

INSPIRATORY MUSCLE STRENGTH (MIP)

A. Leave-one-out sensitivity analysis

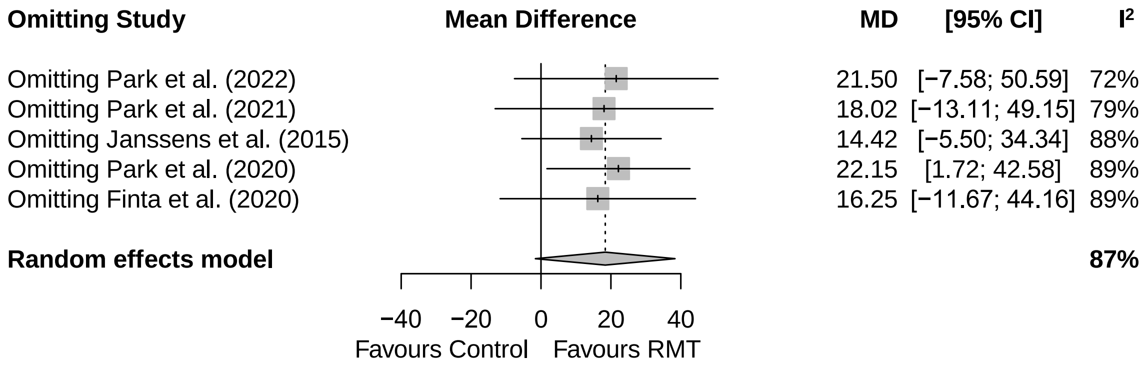

B. Publication bias

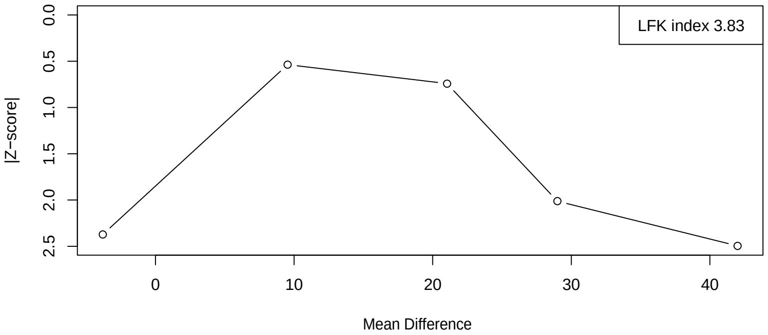

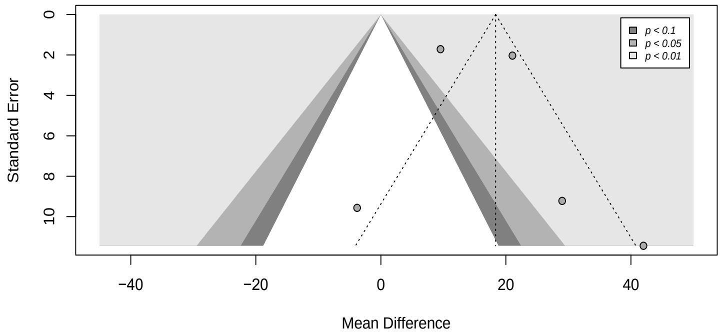

EXPIRATORY MUSCLE STRENGTH (MEP)

A. Leave-one-out sensitivity analysis

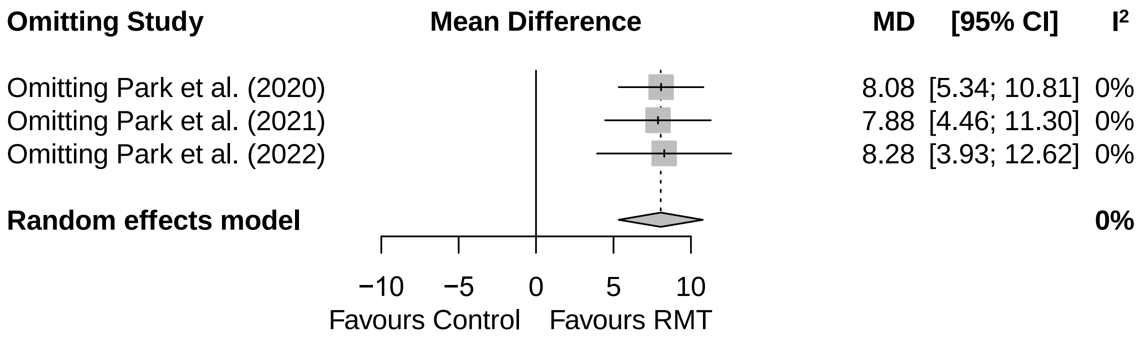

B. Publication bias

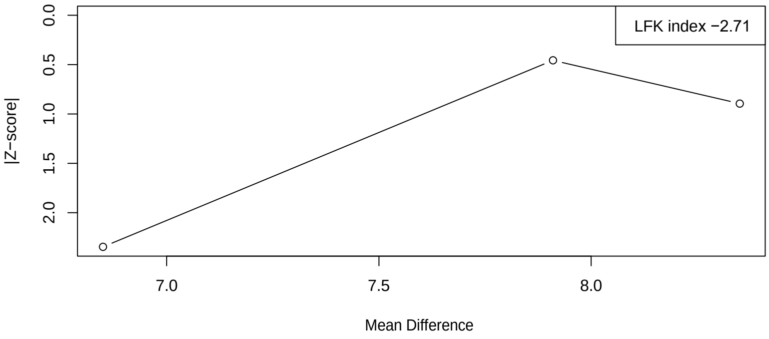

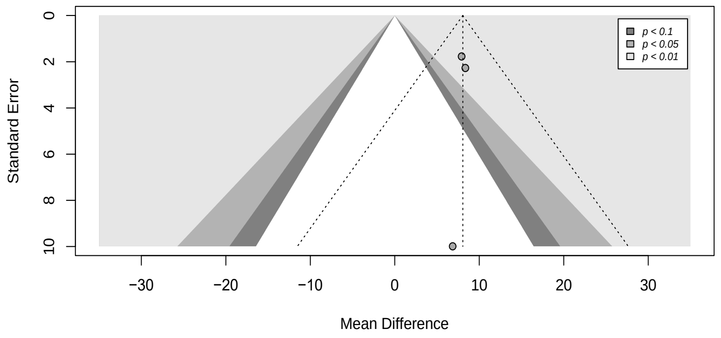

FORCED VITAL CAPACITY (FVC in liters)

A. Leave-one-out sensitivity analysis

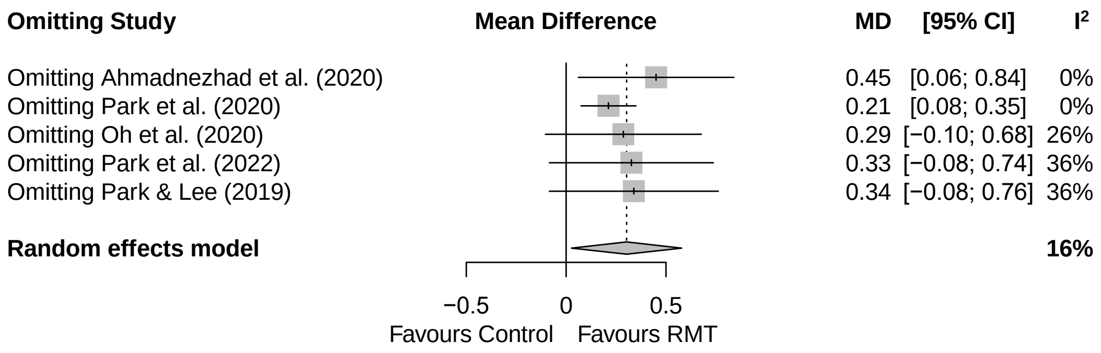

B. Publication bias

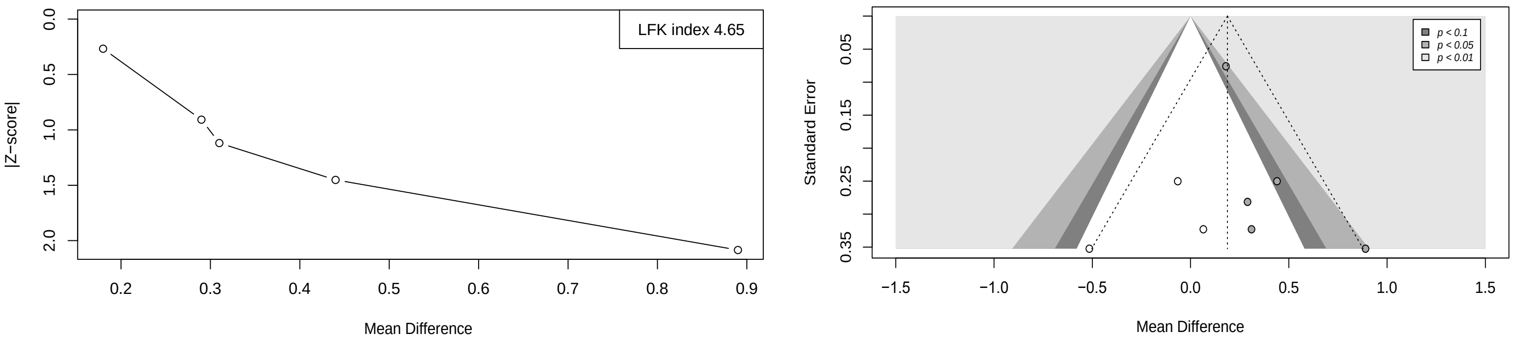

C. Funnel plot of the studies included in the analysis and the studies filled to adjust for publication bias

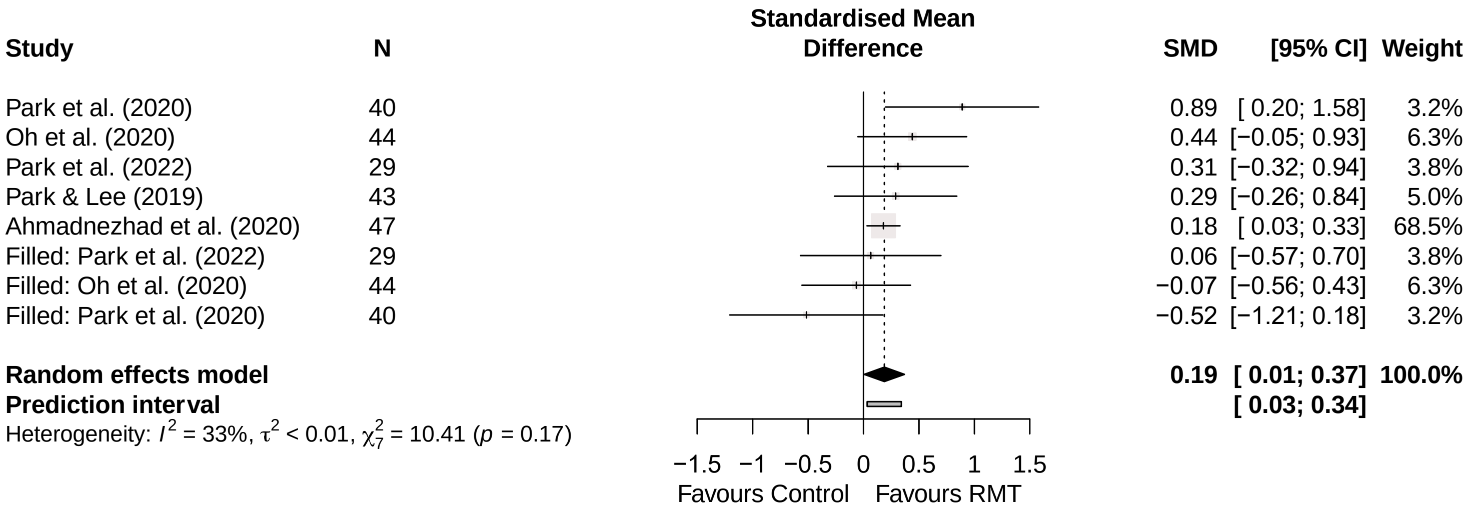

FORCED EXPIRATORY VOLUME IN 1 SECOND (FEV_1 in liters)

A. Leave-one-out sensitivity analysis

Omitting Study	MD	[95% CI]	I^2
Omitting Ahmadnezhad et al. (2020)	0.52	[0.12; 0.92]	0%
Omitting Park et al. (2022)	0.25	[−0.09; 0.59]	19%
Omitting Oh et al. (2020)	0.32	[−0.21; 0.86]	50%
Omitting Park & Lee (2019)	0.40	[−0.18; 0.97]	61%
Omitting Park et al. (2020)	0.39	[−0.17; 0.94]	62%
**Random effects model**			**50%**

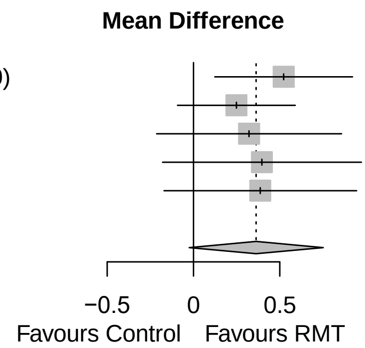

B. Publication bias

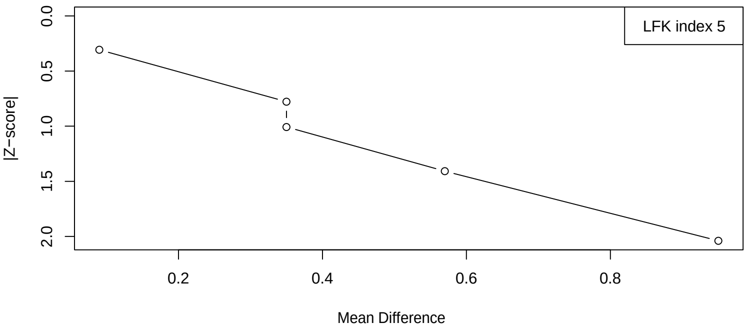

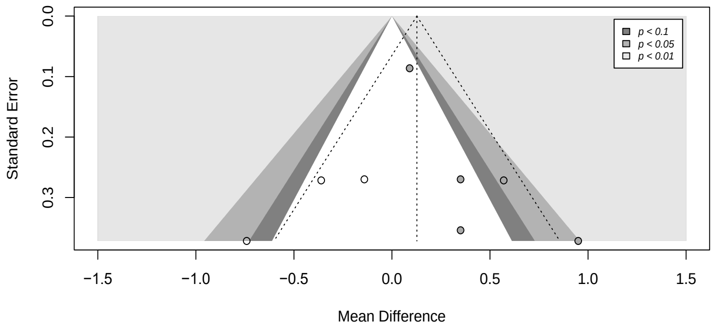

C. Funnel plot of the studies included in the analysis and the studies filled to adjust for publication bias

Study	N	SMD	[95% CI]	Weight
Park et al. (2022)	29	0.95	[0.22; 1.68]	9.8%
Oh et al. (2020)	44	0.57	[0.04; 1.10]	12.8%
Park & Lee (2019)	43	0.35	[−0.18; 0.88]	12.9%
Park et al. (2020)	40	0.35	[−0.34; 1.04]	10.3%
Ahmadnezhad et al. (2020)	47	0.09	[−0.08; 0.26]	18.7%
Filled: Park & Lee (2019)	43	−0.14	[−0.67; 0.39]	12.9%
Filled: Oh et al. (2020)	44	−0.36	[−0.89; 0.17]	12.8%
Filled: Park et al. (2022)	29	−0.74	[−1.47; −0.01]	9.8%
**Random effects model**		**0.13**	**[−0.29; 0.54]**	**100.0%**
**Prediction interval**			**[−0.86; 1.12]**	

Heterogeneity: $I^2 = 62\%$, $\tau^2 = 0.14$, $\chi^2_7 = 18.38$ ($p = 0.01$)

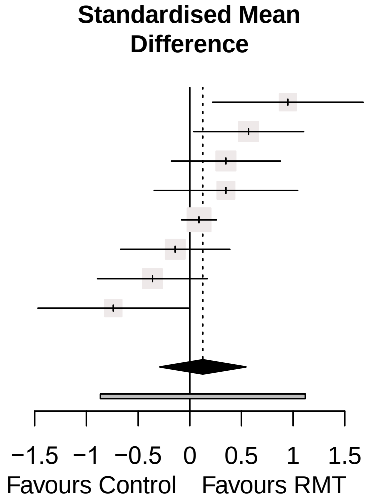

RATIO FEV_1 / FVC (%)

A. Leave-one-out sensitivity analysis

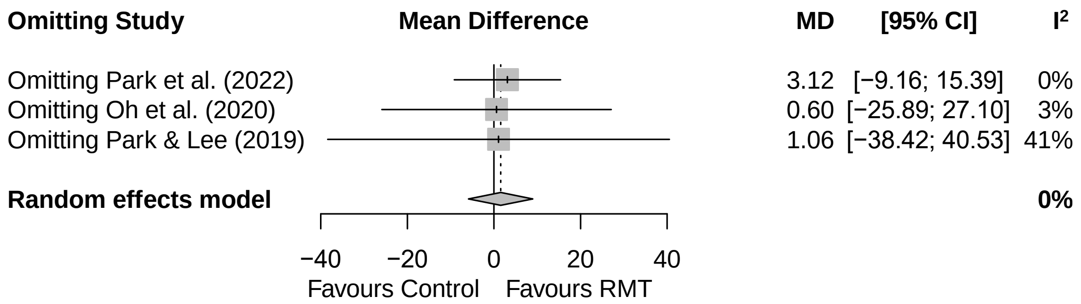

B. Publication bias

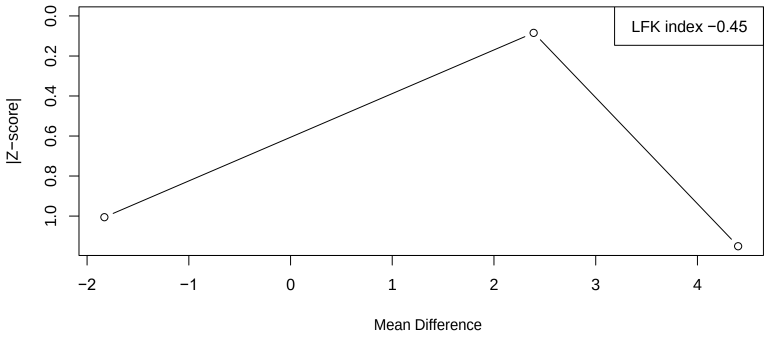

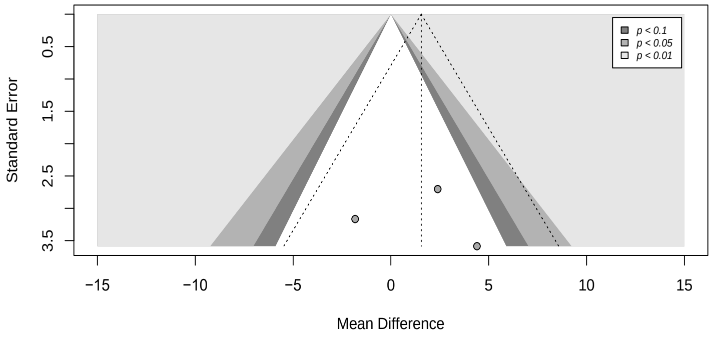

Supplement: Supplementary file 1 [file jcm-13-03053-s001.zip › Figure S4. Sensitivity Respiratory function.pdf]
